# Shoulder pain: to image or not to image?

**DOI:** 10.3389/fresc.2025.1624056

**Published:** 2025-08-29

**Authors:** Fabrizio Brindisino, Paul Salamh, Chad Cook, Jeremy Lewis, Alvisa Palese, Germano Guerra, Jacopo Bonavita, Giacomo Rossettini

**Affiliations:** ^1^ Department of Medicine and Health Science “Vincenzo Tiberio”, University of Molise, Campobasso, Italy; ^2^ Krannert School of Physical Therapy, University of Indianapolis, Indianapolis, IN, United States; ^3^ Department of Rehabilitation Sciences, Tufts School of Medicine, Boston, MA, United States; ^4^ Department of Orthopaedic Surgery, Duke University, Durham, NC, United States; ^5^ Duke Clinical Research Institute, Duke University, Durham, NC, United States; ^6^ Department of Population Health Sciences, Duke University, Durham, NC, United States; ^7^ Therapy Department, Central London Community Healthcare National Health Service Trust, Finchley Memorial Hospital, London, United Kingdom; ^8^ School of Health Sciences, University of Nottingham, Nottingham, United Kingdom; ^9^ School of Life and Health Sciences, University of Nicosia, Nicosia, Cyprus; ^10^ School of Allied Health, University of Limerick, Limerick, Ireland; ^11^ Department of Medical Sciences, University of Udine, Udine, Italy; ^12^ Neurorehabilitation Unit, Villa Rosa Hospital, APSS Trento, Trento, Italy; ^13^ School of Physiotherapy, University of Verona, Verona, Italy; ^14^ Department of Physiotherapy, Faculty of Medicine, Health and Sports, Universidad Europea de Madrid, Madrid, Spain

**Keywords:** diagnostic imaging, ultrasonography, magnetic resonance imaging, clinical relevance, evidence-based practice, practice patterns, physicians'

## 1 Introduction

Imaging findings should be interpreted within the broader context of an individual's shoulder symptoms (1). While imaging is valuable in identifying specific structural pathologies, findings are often equivocal. Defining what imaging changes constitute “normal”, “abnormal”, “unrelated”, “solely causative” findings, and which are, “contributory”, or “associated” with symptoms is a clinical minefield.

Although a naive interpretation might equate “normal” with the absence of pathological features (e.g., no rotator cuff tears, calcification, or labral lesions), this is an oversimplification. For example, rotator cuff tendon tears are frequently observed in individuals without symptoms, who function for years at a very high level of performance. Rotator cuff tendon tears increase asymptomatically with increasing age, suggesting, like wrinkles and grey hair, the tears are likely to be a normal non-noxious senescent change (2). Furthermore, individuals may exhibit full mobility, exceptional muscle performance, no symptoms, and above average function despite observable labral tears, tendon irregularities commonly termed tendinosis, or partial/full-thickness rotator cuff tears (3–7). Moreover, non-sinister and non-traumatic soft tissue imaging considered to be abnormal cannot reliably distinguish currently symptomatic shoulders, previously symptomatic, or those that have never been symptomatic, as the prevalence of “abnormal” findings is often similar across these groups (3, 8, 9). It is arguable that many so called “abnormalities” have been labeled as such because they represent deviations in structure from idealized and flawless anatomical drawings.

The equivocal association between imaging changes and symptoms has lead researchers and clinicians, to question a biomechanical role in symptom causation and/or perpetuation. This is evident in the arguments such as nociception is not needed to experience pain (10), and that pain should be considered a perception and not a sensation (11). These arguments, commonplace in pain science, are not supported by definitive research and should still be regarding as hypotheses and not “fait accompli”. Without doubt, psychosocial factors and the social determinants of health play a seismic role in the experience, perpetuation, and prognosis of symptoms for those living with shoulder pain (12, 13). However, arguments have been made to reframe the relevance of “bio” in shoulder symptoms, and recently a strong case has been made to consider *bio*-chemical factors in the development and perpetuation of rotator cuff related shoulder pain (14).

The appropriate utilization and necessity of shoulder imaging is also equivocal. Guidelines offer inconsistent recommendations and conflicting advice concerning the prescription of radiographs for diagnosing rotator cuff tendinopathy, as some guidelines recommend radiographs during the initial evaluation (e.g., routinely), although others suggest that radiographs *might* be considered, especially when conservative treatment fails (15). Notably, one high-quality guideline did not recommend radiographs for the initial management (15). Lastly, International Consensus suggests using certain *dynamic* imaging techniques as complementary to medical history and physical examination for the clinical assessment of dysfunctional disorders (e.g., subluxation, instability). This approach could aid clinicians in perceiving “functional” disorders rather than solely anatomical or structural injuries (16). As a consequence of this ambiguity, a significant number of clinicians were responsible for referrals for “low-value” imaging, especially for those without a traumatic onset (17).

To promote consideration for the role of “bio” in shoulder symptoms and encourage debate among clinicians and researchers, this opinion paper aims to stimulate discussion on the value of imaging in management of musculoskeletal shoulder pain. We sought to capture diverse perspectives from various health disciplines by incorporating input from clinicians, researchers, educators, physiotherapists, physicians, and nurses working within the musculoskeletal field, aimed at providing a comprehensive perspective to our manuscript.

## 2 Pros and cons of imaging use in shoulder pain management

Although the judicious requests of imaging may improve clinician understanding of a specific clinical scenario, between 20%–50% of imaging requests are inappropriate or of “*low-value”* (e.g., a service offering no or minimal benefit to patients). *Primum non nocere* (first, do no harm) is a core principal in healthcare practice. Unnecessary imaging has the potential to cause harm (e.g., unnecessary risk due to exposure to ionizing radiation) (18), prolong waiting times, and further negatively impact of spiraling healthcare costs (17). Conversely, strategies aimed at reducing low-value imaging have the potential to decrease costs by as much as 95% without compromising patient well-being (17).

How *patients interpret* the imaging report findings will influence their beliefs about appropriate management, (e.g., “I have a tear, I need surgery”) (19). When writing an imaging report, pathoanatomical labelling without reference to the incidence and prevalence of findings in people without symptoms could lead to “medical overuse” as unnecessary injections, surgical and non-surgical procedures (20), as well as healthcare-seeking behavior reinforcing maladaptive beliefs about damage (21).

A further concern in the interpretation of shoulder imaging is the observed fair to moderate inter-rater agreement *among different examiners* when reporting on identical scans (9, 22), a factor that is also influenced by the experience of the individual professional and which can potentially result in patient disorientation, unhelpful guidance, and unwarranted subsequent investigations (23).

Studies investigating this issue, question the *clinical utility* of routine imaging for treatment planning, given the high prevalence of anatomical variations in asymptomatic shoulders (1, 8, 9, 24). Indeed, while imaging may identify tissue pathology, it often cannot reliably determine the clinical significance of these findings or their correlation with specific symptoms. For instance, routine plain radiographs in individuals with atraumatic shoulder pain altered the diagnosis based on medical history and physical examination in fewer than 15% of cases, and clinical management was modified in only 1.7% of cases (25). Furthermore, in individuals with frozen shoulder, additional pathologies were identified via magnetic resonance imaging in 22% of subjects. However, a modification in the treatment plan based on these findings was observed in only 2.7% of cases, and 37 resonance scans were required to identify one patient with frozen shoulder necessitating surgery due to the additional imaging findings (26).

In conclusion, acceptance of imaging findings (i) without defining what abnormal is based upon and (ii) without stating the prevalence of such findings in people without symptoms, may lead to over medicalization and detrimentally impact on healthcare sustainability.

### 2.1 Getting the balance right

The thoughtful application of imaging strategies may confer several advantages. Firstly, imaging may identify relevant structural pathologies when *serious pathology* is a primary concern (27). In complex, traumatic, or suspicious cases, where differential diagnosis is challenging due to the overlap of clinical signs and symptoms, the selection of the appropriate imaging modality may help prevent delays in management (28). Moreover, imaging may corroborate *structural healing* following surgical intervention. For instance, in rotator cuff repair, an ultrasound scan or magnetic resonance imaging can confirm the secure placement of bone anchors, demonstrating the technical success of the surgery. Although importantly, from a clinical standpoint, tendon to tendon, and tendon to bone healing is not a prerequisite for a reduction in pain and improvement in function after repair (29, 30).

When indicated based on a suspicion of *clinically significant structural failure*, imaging will be invaluable for a detailed assessment of tissue biology (e.g., a bony Bankart lesion following trauma-related anterior shoulder instability). This information will guide targeted and shared treatment decisions and facilitate specialist involvement (e.g., surgical consultation) as necessary, promoting a positive multidisciplinary approach involving specialists capable of accurate, evidence-based interpretation of structural details.

Lastly, in cases where non-surgical management does *not result in the expected clinical improvement*, the contribution of traumatically and non-traumatically acquired structural variations warrants evaluation, facilitated by the wise selection of imaging for changing the path of care (27). In Table 1*,* we presented scenarios taken from everyday clinical practice to stimulate discussion on pro- and cons- for imaging use in shoulder pain management, while Supplementary Figure S1 (Supplementary Material*)*, details a SWOT -strength, weaknesses, opportunities, and threat- analysis for the use of imaging in shoulder pain management. Lastly, we have included a clinical decision-making diagram (Supplementary Figure S2, Supplementary Material) to illustrate the authors' proposed method for integrating clinical and imaging findings. This diagram is designed to simplify the decision-making process for determining when imaging is necessary, unnecessary, or a borderline case.

**Table 1:** Clinical practice scenarios to stimulate discussion on pro- and cons- for imaging use in shoulder pain management.

Clinical condition	Clinical scenario	Case discussion
Suspected red flag conditions	*A 56-year-old housewife woman presented* via *direct access with atraumatic right shoulder pain. The patient reported concurrent right thoracic pain (in the region of the 7th rib) and exacerbation of shoulder pain with vigorous housework. She also reported a spontaneous cough of two weeks' duration. Notably, her night pain lessened when lying on her right side. No deficits in strength or sensation were reported, and the clinical shoulder examination was unremarkable*	**Key Clinical Features:** The initial screening allows physiotherapists to guide their decision to either treat, treat and refer for further imaging or consultation, or directly refer the patient for external medical evaluation if the issue are suspected to fails outside the scope of physiotherapy (33, 38). The patient presents with shoulder pain, a concurrent cough, and pain that worsens with exertion but is not reproducible during a physical shoulder examination. The pain is relieved by lying on the affected side. **Decision Point & Rationale:** The combination of symptoms—especially the presence of a cough and pain that doesn't originate from the shoulder joint itself—raises red flags for a non-musculoskeletal cause (39). These signs suggest a potential underlying pulmonary issue, such as pneumonia, pleuritis, or a Pancoast tumor. This suspicion prompts an immediate referral for medical consultation. In fact, shoulder pain can often be referred from various thoracic and visceral pathologies, mimicking a direct shoulder issue (28, 38, 40, 41). **Imaging & Management Impact:** An x-ray is the appropriate initial imaging modality to rule out lung involvement. This timely imaging and referral are crucial for ensuring the patient receives a prompt and accurate diagnosis, leading to appropriate medical treatment rather than ineffective physiotherapy.
Post-traumatic painful shoulder	*A 48-year-old male lawyer presented to a private physiotherapy practice with left shoulder pain. He reported a fall from a height of one meter one week prior. He did not seek emergency department care initially as the pain was not severe; however, the pain has since intensified and is now present at night. Shoulder movements, particularly abduction, were reported as painful. The patient has a history of smoking since the age of 20 years and rheumatoid arthritis diagnosed at age 28, managed with corticosteroids during symptom flare-ups*	**Key Clinical Features:** The patient has a history of trauma, and over the course of a week, their shoulder pain and loss of function have progressively worsened, with notable nocturnal pain. A significant risk factor is their 20-year history of corticosteroid use, which increases fracture risk. The patient also presents with limited external rotation and abduction, which could indicate a posterior shoulder dislocation. **Decision Point & Rationale:** The combination of trauma, progressive pain (especially nocturnal), and the risk factor of long-term steroid use raises a strong suspicion for a fracture (such as an impacted greater tuberosity fracture) or a posterior shoulder dislocation, which can be easily missed (73.2% of cases). Given that these injuries require specific management and can worsen with a delay in diagnosis, immediate imaging is warranted over conservative care. **Imaging & Management Impact:** Imaging is crucial for a definitive diagnosis, and is mandatory when the bone profiles of the clavicle or acromion raise concerns about bone integrity. It is also necessary when the patient experiences severe pain that doesn't decrease even after pharmacological therapy, or when a large hematoma (Hennequin sign) is evident in the anterolateral part of the shoulder and arm. Notably, some fractures like an impacted greater tuberosity fracture, might not cause intense immediate pain and could be not easily recognizable. In this particular scenario, imaging helps to either confirm a fracture or identify a posterior shoulder dislocation, when clinical examination is not enough. In fact, while observable signs like a posterior protuberance, a flattening of the anterior shoulder profile, and coracoid prominence (42) along with anterior and posterior shoulder pain and limited external rotation and abduction [possibly due to an associated McLaughlin lesion (43)] can raise suspicion; these signs might be absent in more corpulent patients. In this case, true antero-posterior and Y-view x-ray projections are mandatory for assessing humeral head position relative to the glenoid (44) and identify this pathology, as it often requires a different course of treatment (e.g., immobilization or surgery) to restore function.
Rotator cuff calcific tendinopathy	*A 54-year-old office worker female presented to a private physiotherapy practice reporting a sudden onset of intense and intolerable pain (Numeric Rating Scale for Pain 8/10) in her shoulder, experienced since the previous day, and an inability to abduct her arm. The patient reported significant nocturnal pain disruption. Her medical history included diabetes and hypothyroidism, but no history of trauma*.	**Key Clinical Features:** Given the clinical features collected during the medical history reporting—middle-aged woman with a history of metabolic/endocrine comorbidities, presenting with sudden, severe nocturnal shoulder pain and restricted active motion- rotator cuff calcific tendinopathy should be considered (45). **Decision Point & Rationale:** The presence of calcific deposits doesn't always correlate with symptoms, as only about half of individuals with calcifications report shoulder pain (46), and the resorption phase of calcification is often linked to increased symptom severity. Clinical examination alone may not reveal the calcification phase (47–49); however, imaging is necessary to confirm the diagnosis and guide a treatment plan (50). **Imaging & Management Impact:** Ultrasound with Power Doppler is the imaging modality of choice. It can confirm the presence of calcification, determine its size and location, and help assess if it is in an active, painful phase. Moreover, a combination of ultrasound and power Doppler can help determine if calcification is the source of pain, significantly influencing management (46). This information is critical for management, as it allows for specific interventions like extracorporeal shockwave therapy or percutaneous lavage, which are highly effective for this condition. The resulting treatment plan can be precisely tailored to the individual, potentially providing faster relief and better long-term outcomes than general conservative care (48, 51–53). Moreover, since calcific deposits can occur in various anatomical locations, they could necessitate diverse conservative or interventional approaches depending on the specific pattern of calcific tendinopathy (54). Consequently, a rehabilitation program tailored to the individual, integrating both clinical and sonographic findings, is vital for optimizing care and enhancing the patient's quality of life (53). It's worth noting that the relationship between calcification resorption and improved clinical outcomes (pain, function, or treatment success) remains debated and it is still a controversial topic (52).
Antero-inferior glenohumeral dislocation	*A 57-year-old male automotive mechanic presented to a private physiotherapy practice following 20 days of immobilization for a traumatic anteroinferior glenohumeral dislocation. Emergency department physicians confirmed reduction* via *radiography and recommended physiotherapy after three weeks of brace immobilization. The patient's medical history included a 15-year history of diabetes and a 10-year history of smoking (10 cigarettes/day); in family history, a rotator cuff repair was reported for the patient's mother and brother*	**Key Clinical Features:** In patients over 40 who have experienced a traumatic shoulder dislocation, a rotator cuff tear is a plausible concern that requires careful investigation. Post-immobilization pain and stiffness can be significant challenges, as they may mask identifiable strength deficits, potentially leading to a missed diagnosis. Key predisposing factors, such as smoking history (quantity and time) (55), diabetes (56), and genetics (57, 58) should also raise suspicion and warrant further assessment. **Decision Point & Rationale:** The main decision point is whether to consider imaging when clinical limitations (like pain or stiffness) prevent a reliable strength test or when predisposing risk factors are present. A delayed diagnosis can compromise rehabilitation outcomes and lead to larger rotator cuff tears. Therefore, evidence-based physiotherapy practice necessitates a proactive approach to screening in these specific patient populations. **Imaging & Management Impact:** Imaging should be considered whenever a rotator cuff tear is suspected, particularly when clinical assessments are unreliable. A comprehensive understanding of the clinical situation, obtained through imaging, is crucial for preventing a missed or delayed diagnosis. Identifying a tear early allows for a more effective and tailored management plan, which can prevent the tear from worsening and significantly improve rehabilitation outcomes.
Frozen shoulder	*A 58-year-old female architect presented with a 4-month history of daily and nocturnal shoulder pain, unresponsive to pharmacotherapy. The patient reported severe sleep disruption and an inability to abduct or rotate her arm. Passive range of motion was also limited, with an empty end-feel due to intractable pain. Her medical history included diabetes, dyslipidemia, a rheumatological pathology (i.e., lupus erythematosus), and a history of uterine carcinoma three years prior*.	**Key Clinical Features:** It's crucial to consider the differential diagnosis of frozen shoulder, as several pathologies can mimic it. Conditions like neoplasms of the proximal humeral head, glenohumeral arthritis, and avascular necrosis can present with similar symptoms and can't be reliably differentiated through a medical history or physical exam alone (59). A patient's predisposing factors, such as diabetes, dyslipidemia, a history of malignancy, or lupus erythematosus, can be risk factors for both frozen shoulder and these other serious conditions. In this particular case, diabetes and dyslipidemia were recognized predisposing factors for frozen shoulder development (60–62), but diabetes and lupus erythematosus are also risk factors for avascular necrosis (63–65). Moreover, the patient's history of malignancy warrants careful consideration (66). **Decision Point & Rationale:** The main decision point revolves around when to order imaging. While some experts don't support routine imaging for every patient, arguing it's unnecessary without a history suggestive of a serious disease, others believe it's essential, especially when predisposing risk factors are present (67). The rationale is to either confirm a diagnosis of frozen shoulder or to identify a potentially life-threatening or debilitating condition that requires different, and often more urgent, management. Therapists should remain vigilant and re-evaluate the diagnosis if a patient doesn't improve with treatment or if their symptoms worsen (67). **Imaging & Management Impact:** Imaging is a critical tool in these scenarios. If a neoplasm is identified, it could be life-saving. If avascular necrosis is diagnosed, prompt and appropriate treatment can significantly improve the patient's quality of life. A recent international consensus suggests that if a patient with a presumed frozen shoulder doesn't respond to initial therapy and an intra-articular injection, imaging should then be performed to rule out other pathologies (68).
Rotator cuff- related shoulder pain	*A 53-year-old male middle school educator presented at a private physiotherapy clinic with a two-week history of anterolateral shoulder pain on the dominant side, representing the first such episode in his lifetime. The patient reported no history of trauma. The individual does not smoke or participate in regular exercise. He has a high body mass index Recent medical history included a relocation to a new residence three weeks prior to presentation. This involved considerable lifting and carrying.* *Clinical examination revealed a painful arc of motion during shoulder abduction; however, a full range of motion was otherwise preserved. Furthermore, strength assessment of external and internal rotation demonstrated comparable mean values bilaterally, while isometric resisted elevation at 90° of arm flexion exhibited diminished strength on the affected side.*	**Key Clinical Features:** The patient presents with shoulder pain but without a history of trauma or intense sporting activity. Key findings include a high body mass index, a history of overuse, minimal restriction in passive range of motion, a painful arc, and reported weakness with abduction. Such factors increase the suspicion of rotator cuff-related shoulder pain (69, 70). **Decision Point & Rationale:** Given the patient's history and symptoms, imaging is not immediately necessary. Instead, the focus should be on a trial of conservative care. Contrariwise, early referral for imaging here, could be disadvantageous. Imaging findings have demonstrated a weak association with symptom perception and are unlikely to alter the treatment trajectory. Moreover, a lay interpretation of imaging reports by non-experts —without adequate education regarding the prevalence and incidence of tendon structural abnormalities in asymptomatic individuals (6, 9)— could be detrimental to engagement in rehabilitation and adherence to therapeutic exercise. Additionally, this could foster expectations for more invasive treatments (19, 21), increase “medical overuse” and reinforce maladaptive beliefs (20, 21). Finally, imaging has not consistently shown prognostic value (12, 13). **Imaging & Management Impact:** A well-structured rehabilitation program is the primary management strategy. This includes patient education on their condition, therapeutic exercises, and lifestyle modifications (e.g., improving sleep, diet, and physical activity) (71–73), delivered through patient-centered communication and incorporating behavioral change strategies into the educational component (74). Exercise is frequently effective, expected, and generally well-received by individuals with rotator cuff-related shoulder pain (75, 76), and lifestyle modifications represent a promising group of interventions for enhancing clinical outcomes in shoulder tendinopathy, (20, 72, 73, 77). Imaging should be reserved for cases where the patient's symptoms don't improve with conservative care or if there's an unexpected worsening of symptoms within a reasonable timeframe (27, 32). This approach avoids “medical overuse,” reduces maladaptive beliefs about the condition, and promotes patient engagement in a proven, effective treatment plan.

## 3 Implications

Prior to, or during attendance at out-patient shoulder orthopedic clinics routine imaging (usually radiographs) is common practice. This may help identify rare conditions requiring urgent management, such as osteosarcomas. However, in-depth interview practice and appropriate safety netting would reduce the need for blanket imaging and lessen the potential harms associated with being informed of tendon and/or bone changes that may, and equally may not be associated with symptoms and in the main will respond equally well to non-surgical management.

“Low-value” imaging equates to billions of dollars of wasted resources. For example, within magnetic resonance imaging, a primary cohort receiving imaging referrals discordant with guidelines included orthopedic patients and patients referred by general practitioners (17, 31). Additionally, atraumatic pain was identified as a specific clinical condition where the use of “low-value” imaging was particularly evident (17). Measures for reducing “low-value” imaging and to promote reasonable use and recommendation of it are needed at all levels of healthcare, for promoting interprofessional collaboration and networking, thereby improving patient care and perceived support by the patient.

Clinical guidelines recommend judicious use of imaging in people with shoulder pain, discouraging routine imaging, but fully utilize their use in probable red flag presentations (27, 32). Imaging should also be considered in non-improving (possibly at 12 weeks), or earlier, with worsening symptoms (27, 32). The appropriate utilization of imaging for a comprehensive understanding of clinical scenarios holds significant implications for both clinical practice and research. Clinicians (e.g., physicians, chiropractors, physiotherapists) bear the responsibility to justify the need to image and if a decision to image is made, determine the appropriate timing and type of imaging studies. This must be done within a shared decision making framework, and to recognize when multidisciplinary consultation is necessary to address patient needs effectively (33).

Shared decision making requires consideration of the benefits and potential harms of imaging. It needs to be framed within comprehensive patient education, encompassing the current clinical status and patient wishes for management. For example, in the absence of red flag concerns is imaging necessary for a patient who wishes to focus on non-invasive, non-surgical management (34)?

This opinion paper aims to stimulate a *mature, balanced discourse* that empowers less experienced clinicians to make informed decisions with individual patients, considering all pertinent factors contributing to their well-being. Within a proper application of the bio-psycho-social model of care, biological variables must be acknowledged, and in-depth analysis through imaging modalities can serve as a valuable strategy. As showed in the six clinical cases in Table 1, people presenting with painful shoulder conditions, particularly when specific anamnesis and predisposing factors were present, imaging may:

• Confirm the presence of structural abnormalities requiring multidisciplinary consultation [e.g., expert opinion(s)].
• Guide clinicians towards targeted interventions addressing specific structural pathologies, thereby enhancing treatment efficacy (e.g., calcific tendinopathy).
• Prevent adverse outcomes resulting from misdiagnosed conditions (e.g., instability with rotator cuff tear).
• Facilitate accurate differential diagnosis, mitigating the risk of overlooking clinically significant comorbidities (e.g., frozen shoulder).

## 4 Conclusion

Clinical practice frequently lacks balance and frequently is conducted at the ends of a spectrum. With one end being rigid reliance on structural imaging and the other its rejection in favor of a psychosocial narrative. Yet clinical reality is rarely binary, and practicing at the end of any spectrum, as is in most situations in life, leads to cognitive distortion, and blind adherence to one belief over another. Recent medical examples include the discourse on vaccination, and in physiotherapy, the -often- toxic debate relating to touch therapy. The Latin phrase, *in medias res* (the solution lies in the middle) is very apt.

To promote a judicious, resourceful, and evidence-based utilization of imaging, research should emphasize a comprehensive bio-psycho-social care pathway. Within this framework, all relevant domains should be engaged and weighted according to the specific clinical context.

Educational curricula for all health professions should incorporate formative modules designed to enhance expertise in imaging strategies, including their clinical applicability and utility, strengths and limitations, and potential benefits and drawbacks. Moreover, receiving individualized written audit and feedback on imaging request rates —including the benefits of addressing imaging overuse, links to educational resources, and guidance on limiting imaging requests— could significantly decreased overall rate of musculoskeletal imaging requests (35–37), and could be a widely strategy for enhancing professional practice.

A balanced, context-driven use of imaging, grounded in clinical reasoning and embedded in a biopsychosocial model, can enhance diagnosis, inform treatment, and support shared decision-making. Imaging is a valuable tool when used wisely: the question is not whether to use it, but when, why, and for whom.
