# Supplementary figures and images for: Shoulder pain: to image or not to image?
Source: Front Rehabil Sci. 2025 Aug 29;6:1624056. doi: 10.3389/fresc.2025.1624056 (PMC12425999; doi:10.3389/fresc.2025.1624056)

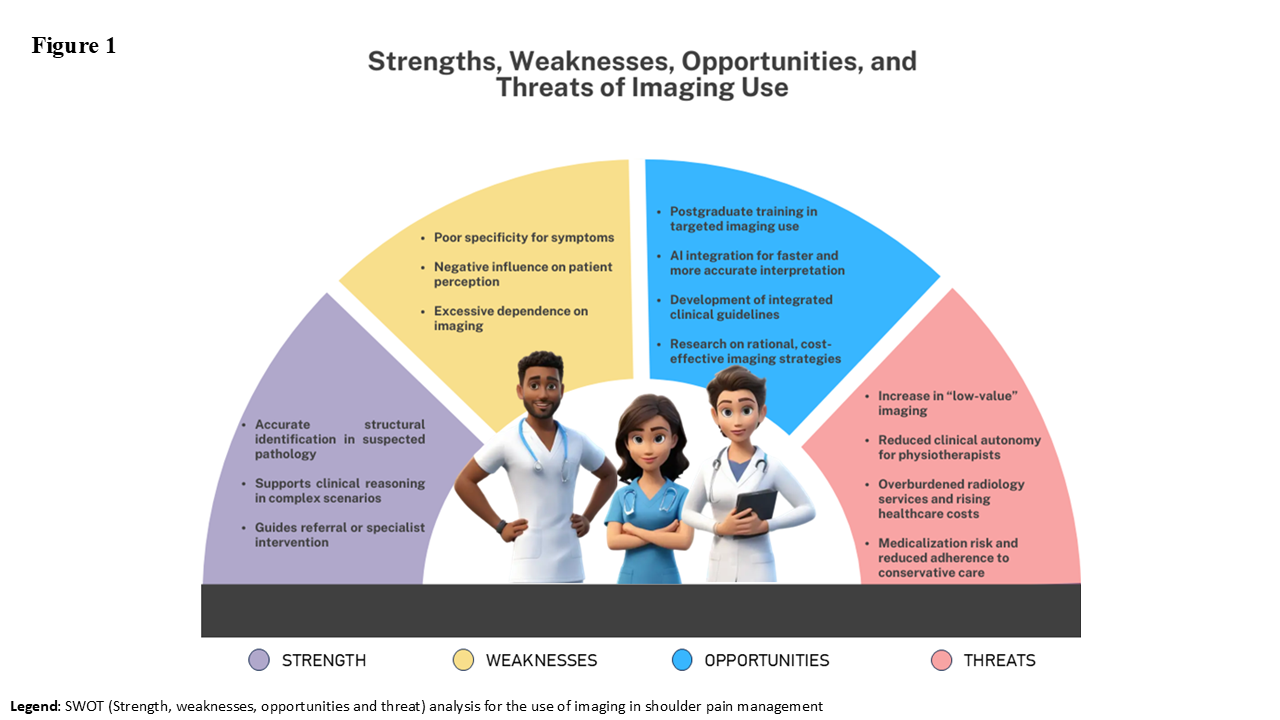

Supplement: Supplementary file 1 [file Image1.tif]

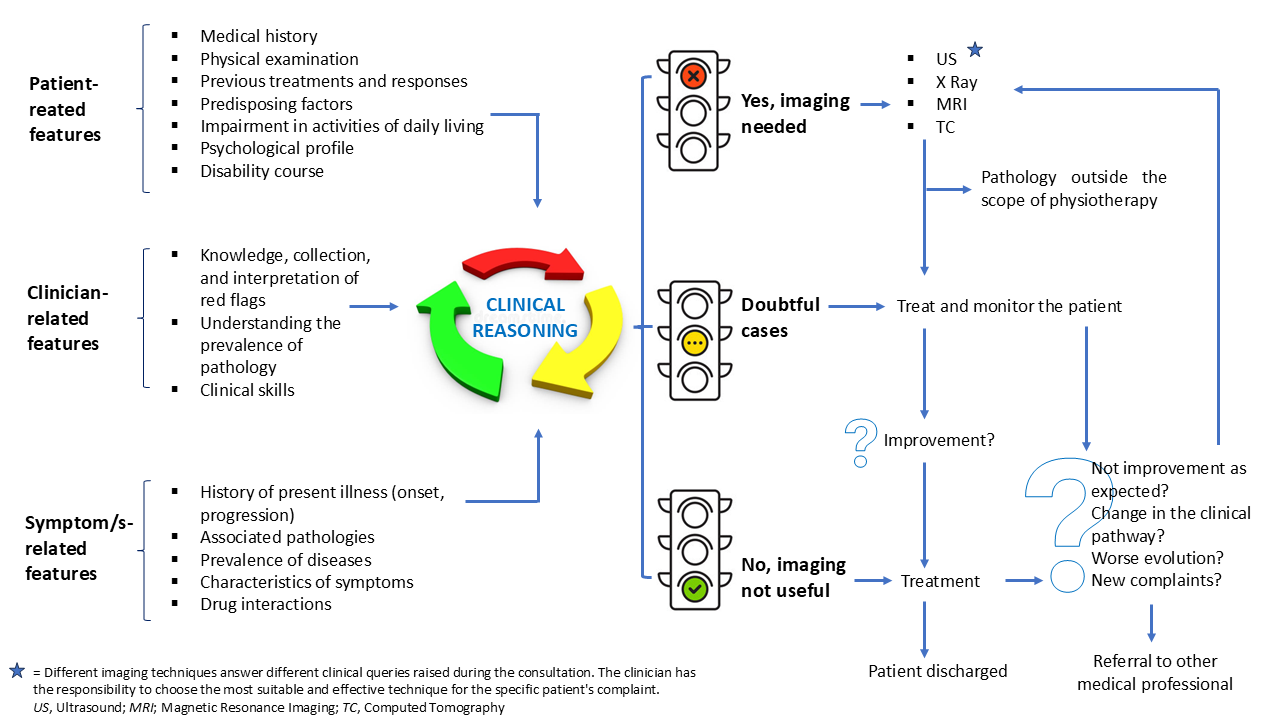

Supplement: Supplementary file 2 [file Image2.tif]
